# Indirect cost of maternal deaths in the WHO African Region in 2010

**DOI:** 10.1186/1471-2393-14-299

**Published:** 2014-08-31

**Authors:** Joses Muthuri Kirigia, Germano Mwige Mwabu, Juliet Nabyonga Orem, Rosenabi Deborah Karimi Muthuri

**Affiliations:** Research, Publications and Library Services Programme, Health Systems and Services Cluster, World Health Organization Regional Office for Africa, Brazzaville, Congo; School of Economics, University of Nairobi, Nairobi, Kenya; Health Systems and Services Cluster, World Health Organization Regional Office for Africa, Brazzaville, Congo; Department of Psychology, School of Social Sciences and Humanities, United States International University, Nairobi, Kenya

**Keywords:** African Region, Maternal mortality, Indirect costs, Loss in non-health GDP

## Abstract

**Background:**

An estimated 147,741 maternal deaths occurred in 2010 in 45 of the 47 countries in the African Region of the World Health Organization (WHO). The objective of this study was to estimate the indirect cost of maternal deaths in the Region to provide data for use in advocacy for increased domestic and external investment in multisectoral policy interventions to curb maternal mortality.

**Methods:**

This study used the cost-of-illness method to estimate the indirect cost of maternal mortality, i.e. the loss in non-health gross domestic product (GDP) attributable to maternal deaths. Estimates on maternal mortality for 2010 from *Trends in maternal mortality: 1990 to 2010* published by WHO, UNICEF, UNFPA and the World Bank were used in these calculations. Values for future non-health GDP lost were converted into their present values by applying a 3% discount rate. One-way sensitivity analysis at 5% and 10% discount rates assessed the impact on non-health GDP loss. Indirect cost analysis was undertaken for the countries, categorized under three income groups. Group 1 consisted of nine high and upper middle income countries, Group 2 of 12 lower middle income countries, and Group 3 of 26 low income countries. Estimates for Seychelles in Group 1 and South Sudan in Group 3 were not provided in the source used.

**Results:**

The 147,741 maternal deaths that occurred in 45 countries in the African Region in 2010 resulted in a total non-health GDP loss of Int$ 4.5 billion (PPP). About 24.5% of the loss was in Group 1 countries, 44.9% in Group 2 countries and 30.6% in Group 3 countries. This translated into losses in non-health GDP of Int$ 139,219, Int$ 35,440 and Int$ 16,397 per maternal death, respectively, for the three groups. Using discount rates of 5% and 10% reduced the total non-health GDP loss by 19.1% and 47.7%, respectively.

**Conclusion:**

Maternal mortality is responsible for a noteworthy level of non-health GDP loss among the countries in the African Region. There is urgent need, therefore, to increase domestic and external investment to scale up coverage of existing cost-effective, multisectoral women’s health interventions to reduce maternal morbidity and mortality.

**Electronic supplementary material:**

The online version of this article (doi:10.1186/1471-2393-14-299) contains supplementary material, which is available to authorized users.

## Background

Maternal mortality refers to the death of a woman during pregnancy or childbirth or within 42 days after delivery [1]. Out of the 287,000 maternal deaths that occurred globally in 2010, some 147,741 (51%) were borne by countries in the African Region of the World Health Organization (WHO) [2]. About 28% of the deaths in the African Region resulted from haemorrhage, 14% from abortion, 12% from sepsis, 9% from hypertension disorders and 5% from obstructed labour. About 37% were associated with other conditions such as HIV, malaria or diabetes [3]. The African Region’s maternal mortality ratio (MMR) of 480 per 100,000 live births was almost 25 times that of European Region of WHO [4].

The United Nations’ fifth Millennium Development Goal (MDG 5) aims to improve maternal health to give impetus to country efforts to stem the tide of maternal morbidity and mortality. Target 5A of MDG 5 aims at reducing MMR by 75% between 1990 and 2015, which would require an annual decline of at least 5.5%. Target 5B requires countries to achieve universal access to reproductive health services by 2015 [5].

By the end of 2010, out of the 44 countries that reported data, only two were on track to achieve target 5A, meaning that their MMR had declined by 5.5% or more annually. Twenty-six countries were making progress, with MMR declines of 2% to 5.5% per year, nine made insufficient progress with MMR declines of less than 2% annually, and seven made *no progress* (Table 1) [2].

**Table 1:** **Progress of WHO African countries towards MDG5A in 2010 and 2013**

Progress status	Countries in 2010
On track	Equatorial Guinea, Eritrea
Making progress	Algeria, Angola, Benin, Burkina Faso, Cape Verde, Comoros, Cote d’Ivoire, Democratic Republic of Congo, Ethiopia, Gambia, Ghana, Guinea, Liberia, Madagascar, Malawi, Mali, Mauritania, Mozambique, Niger, Nigeria, Rwanda, Sao Tome and Principe, Senegal, Togo, Uganda, Tanzania
Insufficient progress	Burundi, Central African Republic, Gabon, Guinea-Bissau, Kenya, Sierra Leone, South Africa, Swaziland, Zambia
No progress	Botswana, Cameroon, Chad, Congo, Lesotho, Namibia, Zimbabwe

Source: WHO, UNICEF, UNFPA and The World Bank [2].

The fact that so many countries were not on track to achieve MDG 5 targets could be attributed to the poor coverage of maternal health services. For example, only 43% of pregnant women had at least four visits for antenatal care, just 49% of the births had a skilled attendant, contraceptive use prevalence was only 27%, 25% of the family planning services need was unmet, and only 45% of new mothers made postnatal care visits within two days of childbirth [4]. The unfavourable state of maternal health services is largely associated with the a paucity of resources in the health sector in the countries in general [6] and in women’s health specifically [1, 7]. There is need for studies on the total cost of illness to provide data for use by the ministries of health in advocacy with the ministries of finance, development partners and the private sector for more resources for health-promoting interventions and health services geared at preventing maternal morbidity and mortality.

Resolution AFR/RC58/R1 adopted by the fifty-eighth session of the WHO Regional Committee for Africa, requested the WHO Regional Director for Africa to establish an African Commission for Women’s Health to generate evidence on the impact on socioeconomic development of improving the health of women for use to improve advocacy and policy action [8]. Prior to the setting up of that commission, only one study, conducted in 2005, had attempted to estimate the impact of maternal deaths on GDP in the African Region using econometric modelling [9]. That study found that maternal mortality of a single person reduced per capita GDP by US$ 0.36 [9]. The commission deemed that finding to be outdated and a gross underestimation of the economic loss attributable to maternal death.

This paper is an updated version of the contribution the authors made to the report of the African Commission for Women’s Health [10]. It addresses the question: What is the impact of maternal deaths on non-health GDP in the WHO African Region?

The specific objective was to generate estimates of indirect cost of maternal deaths in the WHO African Region in 2010 for use in advocacy for increased investment in mutlisectoral policy interventions to curb maternal morbidity and mortality.

## Methods

### Conceptual framework

Maternal death leads to losses in current and future production, income and consumption of non-health goods and services. The main channels through which maternal deaths affect macroeconomic output include increased health expenditure, labour and productivity losses and reduced investment in human and physical capital formation [11]. The quantity of interest in this study is not the forgone economic welfare associated with market or non-market production but the market production forgone due to maternal deaths.

One of the measures of the market production forgone because of maternal deaths is GDP, that is the total value of all marketed final goods and services produced in the economy during a given period of time (usually a year) [12]. It is the sum of personal consumption expenditures, gross private investment, government consumption spending, and net exports, i.e. exports minus imports.

*Consumption* refers to household and business enterprise spending on goods and services such as health and education services, water, sanitation, clothing, food, sports, movies, books, computers, refrigerators, vehicles, etc. Maternal mortality reduces the number of persons who spend money on such goods and services. Reductions in expenditure due to maternal mortality most likely lead to reductions in the production of consumer goods and services with the decreased demand.

*Investment* refers to expenditure on capital goods from households, private business enterprises, public corporations and public authorities over a given period. Examples of capital goods are residential and non-residential buildings, infrastructure, transport equipment, and machinery and other equipment. Maternal death affects investment by reducing the quantity and quality of skilled, semi-skilled and unskilled female labour available for investors. Also, given that money for domestic investment usually comes from household and firm savings, maternal deaths reduce the number of savers and the quantity of savings, and hence, the resources available for investment.

*Government expenditure* consists of spending by central, provincial and local levels of government on goods such as office supplies and services such as salaries of public service employees and security services. The money that the government spends comes primarily from taxation, and taxes come from household and business incomes. Maternal deaths reduce the number of actual taxpayers and so erode the volumes of current and future tax revenues.

*Net exports* are the difference between exports and imports. A sizeable portion of total production of goods (for example tea, coffee, cotton, palm oil, cocoa, animal products, horticultural products, floriculture products, petroleum, minerals) and services such as tourism is *exported.* A part of total expenditure is used on imported goods and services. Death of women reduces the quantities of exports and, hence, earnings from exports.

In this study, *the quantity of interest* is the impact of maternal mortality on the non-health components of GDP, since the use of health services or goods does not generate utility or welfare per se [13]. Chisholm *et al.* [14] argue that *"…* the quantity of interest cannot be GDP, because medical care and health expenses actually form part of GDP; instead … a more appropriate quantity of interest would be the impact of disease or injury on the non-health components of GDP" (p. 584).

### Calculation of non-health GDP loss due to maternal deaths

A country’s net present value of current and future non-health GDP lost due to maternal deaths is the sum of non-health GDP loss among women aged under 15 years and those within the reproductive age group of 15–49 years. The non-health GDP loss attributable to maternal deaths among persons of a specific age group was estimated by multiplying the total number of discounted years of life lost (which is equivalent to the sum of the discount factors) by the non-health GDP per capita and the total number of maternal deaths in the age group. Discounting renders losses occurring in different time periods comparable by expressing their values in present terms. Thus, discounting measures how much future losses are worth today. One argument for discounting future health is that there exists a small level of uncertainty of survival in a society that increases monotonically over time [15].

Non-health GDP loss due to maternal deaths among women under 15 years of age was estimated by multiplying the total discounted years of life lost by the non-health GDP per capita and the total maternal deaths for women aged under 15 years. For example, in Algeria, 61 undiscounted years of life were lost per a death in the group of women aged under 15 years. This value was obtained by deducting 13, the average age at death, from 74, the female life expectancy. Discounting at 3% yielded 27.84035307 discounted years of life lost. GDP per capita of Int$ 7,957.8 minus per capita total health expenditure of Int$ 364.28 equals Int$ 7611.522 non-health GDP per capita. Total maternal deaths for women aged under 15 years were 6.251182185. Thus, the net present value of non-health GDP lost due to maternal deaths among women aged under 15 years is Int$ 1,324,672.14, which is obtained by multiplying 27.84035307, the discounted years lost, by Int$ 7611.522, the per capita non-health GDP, and the 6.251182185 deaths.

Non-health GDP loss due to maternal deaths among women aged 15–49 was estimated by multiplying the total discounted years of life lost (the sum of discount factors) by the non-health GDP per capita and the total maternal deaths in the 15–49 age group. In Algeria, 44 undiscounted years of life were lost per one death in the 15–49 age group. This value was obtained by subtracting 32 years, the average age at death, from 74, Algeria’s female life expectancy at birth. Discounting at 3% yielded 24.25427392 discounted years of life lost. GDP per capita of Int$ 7,957.8 minus per capita total health expenditure of Int$ 364.28 equals Int$ 7611.522 non-health GDP per capita. Total maternal deaths for women aged 15–49 years were 683.7488178. The net present value of non-health GDP lost due to maternal deaths among those aged 15–49 years is Int$ 126,228,195.41, which is obtained by multiplying the 24.25427392 discounted years lost by Int$ 7611.522, the per capita non-health GDP, and the 683.7488178 deaths. See Table 2 for an illustration of how to calculate the loss in total non-health GDP using actual data from Algeria.

**Table 2:** **Illustration of how to calculate loss in total non-health GDP using actual information on Algeria**

Variables	Values
(a) Algeria total maternal deaths in 2010	690
(b) Proportion of deaths occurring under 15 years	0.00905968432540048
(c) Proportion of deaths occurring 15–45 years	0.9909403156746
(d) *TMD*_<15_ = 690 x 0.00905968432540048	6.251182185
(e) *TMD*_15–45_ = 690 x 0.9909403156746	683.7488178
(f) Average age at death for those dying below 15 years (*AAD*_<15_)	13
(g) Average age at death for those dying between 15–45 years (*AAD*_15–45_), i.e. (15 + 49)/2	32
(h) Algeria average female life expectancy at birth (LE) in years	74
(i) *NHGDPPC* = *GDPPC*_*Int$*_ - *PCTHE* = *Int$* 7,975.80 -Int$364.28	Int$7611.522
(j) Discount rate (*r)*	3%
(k) Undiscounted years of life list by under 15 years *(YLL*_<15_) = LE - *AAD*_<15_ = 74 – 13	61
(l) Discounted years of life lost by under 15 years *(YLL*_<15_)	27.84035307
(m) Undiscounted years of life lost by those aged 15 to 49 year *YLL*_15–45_ = LE - *AAD*_15–49_ = 74–32	44
(n) Discounted *YLL*_15–49_	24.25427392
(o) *NHGDPLoss*_<15_ = Discounted *YLL*_<15_ x *NHGDPPC*_*Int$*_ x *TMD*_<15_ = 27.84035307 x 7611.522 x 6.251182185	Int$1,324,672.14
(p) *NHGDPLoss*_15–49_ = Discounted *YLL*_15–49_ x *NHGDPPC*_*Int$*_ x *TMD*_15–49_ = 24.25427392 x 7611.522 x 683.7488178	Int$126,228,195.41
(q) *TNHGDPLoss* = (*NHGDPLoss*_<15_ + *NHGDPLoss*_15–49_) = Int$1,324,672.14+ Int$ 126,228,195.41	Int$127,552,867.55

Each country’s discounted total non-health GDP loss due to maternal deaths was calculated using equations (1), (2) and (3).
123

Where: *TNHGDPLoss* is the total non-health GDP loss due to maternal deaths in a country; *NHGDPLoss*_<15_ is the total non-health GDP loss due to maternal deaths among those under 15 years; *NHGDPLoss*_15 - 49_ is the total non-health GDP loss due to maternal deaths among the 15–49 age group; *NHGDPPC*_*Int$*_ is the per capita non-health GDP in purchasing power parity (PPP), which is obtained by subtracting per capita total health expenditure (PCTHE) from per capita GDP (GDPPC); 1/(1 + *r*)^*t*^ is the discount factor; *r* is the discount rate;  is the summation from year *t* to *n*; *t* is first year of life lost and *n* is the final year of the total number of years of life lost per maternal death, which is obtained by subtracting the average age at death (AAD) from pregnancy- or childbirth-related causes from each country’s average female life expectancy at birth; *TMD*_<15_ is the total number of maternal deaths that occurred among women under the age of 15 in country *j* in 2010; and *TMD*_15 - 49_ is the total number of maternal deaths that occurred among women aged 15–49 in country *j* in 2010. Per capita non-health GDP in purchasing power parity for each of the 45 WHO African Region countries was obtained by subtracting per capita total health expenditure from per capita GDP.

Each country’s female average life expectancy at birth was used as the upper limit for life when calculating the potential years of life lost [16]. That was preferable to arbitrarily choosing an upper limit higher than the country’s average life expectancy [17].

### Sensitivity analysis

Sensitivity analysis entails varying the parameter values of key assumptions underlying the estimates to assess the robustness of the findings or conclusions. One of the main candidates for sensitivity analysis in our study was the discount rate, since time preference might be important but the degree of importance might not be known. The analysis for this study used a rate of 3% to discount future non-health GDP losses into their present values. That is the rate commonly used in WHO’s global burden of disease estimates [18], health system performance assessment [19] and health-related economic evaluation studies in Africa [20]. Additionally, one-way sensitivity analysis was conducted at 5% and 10% discount rates to ascertain their effect on non-health GDP loss estimates.

### Data sources and analysis

The WHO African Region has 47 Member States. The analysis was undertaken with the countries placed in three economic groups. Group 1 had nine high and upper middle income countries, Group 2 comprised 12 lower middle income countries and Group 3 had 26 lower income countries. Maternal mortality data were not available for Seychelles in Group 1 and South Sudan in Group 3. The analysis reported in this paper is for the 45 countries with complete data (Table 3) [21].

**Table 3:** **Classification of countries according to gross national income per capita, (at US dollars) in 2012**

Group	GNI per capita (US$)	Countries
**Group 1**: High-income & upper middle income	> = $4,086	Algeria, Angola, Botswana, Equatorial Guinea, Gabon, Mauritius, Namibia, Seychelles*, South Africa (9)
**Group 2**: Lower middle income	$1,036-4,085	Cameroon, Cape Verde, Congo, Cote d’Ivoire, Ghana, Lesotho, Mauritania, Nigeria, Sao Tome and Principe, Senegal, Swaziland, Zambia (12)
**Group 3**: Low-income	$1,036 or less	Benin, Burkina Faso, Burundi, Central African Republic, Chad, Comoros, DRC, Eritrea, Ethiopia, Gambia, Guinea, Guinea-Bissau, Kenya, Liberia, Madagascar, Malawi, Mali, Mozambique, Niger, Rwanda, Sierra Leone, South Sudan*, Togo, Uganda, United Republic of Tanzania, Zimbabwe (26)

Source: The World Bank [21]. Note: *Missing maternal mortality data, and thus, not included in the analysis.

All the data used in this study were for 2010 (see Additional file 1: Data inputs). The data used to estimate equations 1, 2 and 3 were obtained from four sources. The information on female life expectancy at birth came from the WHO life tables for Member States [22]. The proportions of maternal deaths for those aged under 15 years and 15–49 years were regional estimates from the WHO disease and injury database [23]. The data for the total maternal deaths for 2010 were from *Trends in maternal mortality: 1990 to 2010* published by WHO, UNICEF, UNFPA and the World Bank report [2]. Per capita gross domestic product in purchasing-power-parity (PPP) for 2010 was from a World Bank database [21]. The 2010 per capita total health expenditure was from World Health Statistics, 2013 [4]. The data collection methodologies for each dataset can be found in the data sources. The algorithm for estimating equations 1, 2 and 3 was built in Excel with a spreadsheet for each of the 45 countries.

## Results and discussion

Table 4 presents the WHO African Region’s population, maternal deaths and total GDP by economic group in 2010. An estimated 147,741 maternal deaths occurred in the Region that year. About 5.3% of these were in the high and upper middle income countries (Group 1), 38.2% in the lower middle income countries (Group 2), and 56.5% in the low income countries (Group 3). The average number of maternal deaths per country was 3,283 (STD = 6,296) with a wide variation among the countries, ranging from four in Sao Tome and Principe to 40,000 in Nigeria. The regional average female life expectancy at birth was 59.5 years (STD = 7.2), with 47 years in Sierra Leone as the minimum and 78 years in Mauritius as the maximum. The average non-health GDP per capita in the Region was Int$ 3,718 (STD = 5625), varying from Int$ 343 in the Democratic Republic of Congo to Int$ 26,792 in Equatorial Guinea.

**Table 4:** **Total population, maternal deaths and gross domestic product by economic group in WHO African region in 2010**

Group/economic class	Total population*	Total number of maternal deaths**	Total GDP in purchasing power parity*
Group 1: High income & upper middle income	114,374,635	7,850	1,031,252,095,322
Group 2: Lower middle income	261,328,194	56,482	583,541,933,185
Group 3 : Low income	469,947,141	83,409	470,816,741,335
GRAND TOTAL	845,649,970	147,741	2,085,610,769,842

Source: *The World Bank [21]. ** WHO, UNICEF, UNFPA and The World Bank [2].

### Non-health GDP loss attributable to maternal deaths

The 147,741 maternal deaths in the African Region in 2010 resulted in a grand total non-health GDP loss of Int$ 4.462 billion (PPP) or about 0.21% of total GDP (see Table 5). About 1.2% of the loss occurred among women aged below 15 years and 98.8% among those aged 15–49 years. Of the grand total loss, 24.5% was borne by Group 1 countries, 44.9% by Group 2 countries and 30.6% by Group 3 countries. The average grand total non-health GDP loss per maternal death was Int$ 30,203.

**Table 5:** **Non-health GDP lost due to maternal deaths in 2010 international dollars at 3%, 5% and 10% discount rates**

	Non-health GDP lost due to maternal deaths in 2010 international dollars at 3% discount rate			
Cost items	Group 1 :	Group 2 :	Group 3 :	Grand total Cost
	Cost (Int$)	Cost (Int$)	Cost (Int$)	(Int$)
(1). Total cost of maternal deaths	1,092,866,973	2,001,723,471	1,367,677,434	4,462,267,878
(2). Average cost per maternal death	139,219	35,440	16,397	30,203
(3). Average cost per person in population	9.6	7.7	2.9	5.3
	**Non-health GDP lost due to maternal deaths in 2010 international dollars at 5% discount rate**			
Cost items	Group 1 :	Group 2 :	Group 3 :	Grand total Cost
	Cost (Int$)	Cost (Int$)	Cost (Int$)	(Int$)
(1). Total cost of maternal deaths	869,540,868	1,639,162,741	1,102,015,811	3,610,719,420
(2). Average cost per maternal death	110,770	29,021	13,212	24,440
(3). Average cost per person in population	7.6	6.3	2.3	4.3
	**Non-health GDP lost due to maternal deaths in 2010 international dollars at 10% discount rate**			
Cost items	Group 1 :	Group 2 :	Group 3 :	Grand total Cost
	Cost (Int$)	Cost (Int$)	Cost (Int$)	(Int$)
(1). Total cost of maternal deaths	548,415,806	1,078,513,191	707,591,700	2,334,520,696
(2). Average cost per maternal death	69,862	19,095	8,483	15,801
(3). Average cost per person in population	4.8	4.1	1.5	2.8

### Non-health GDP loss for Group 1 countries

**Figure 1:**
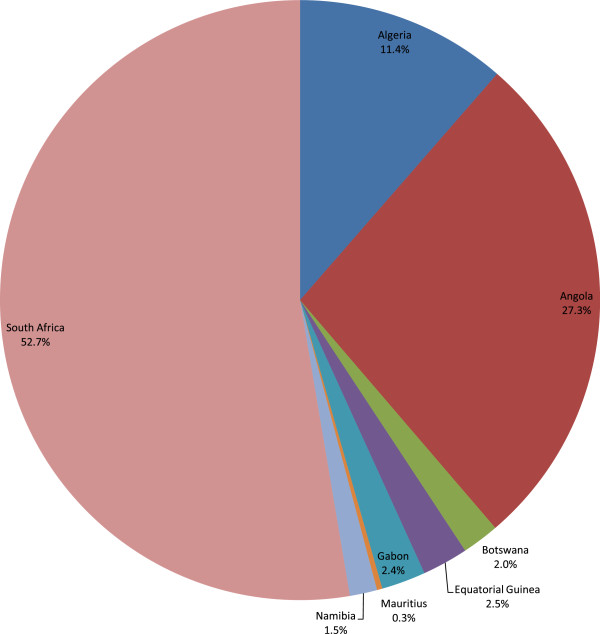
**Group1 - Non-health GDP loss due to maternal deaths in high income & upper middle income countries.**

The 7,850 maternal deaths in Group 1 countries caused a total loss in non-health GDP of Int$ 1,092,866,973 in 2010, or 0.11% of the total GDP for that group. The range was from Int$ 3.1 million in Mauritius to Int$ 575.5 million in South Africa. Figure 1 presents the distribution of Group 1 present values of the total non-health GDP loss (Int$ or PPP) across the eight high and upper middle income countries. About 52.7% of the Group 1 loss was borne by South Africa.

### Non-health GDP loss for Group 2 countries

**Figure 2:**
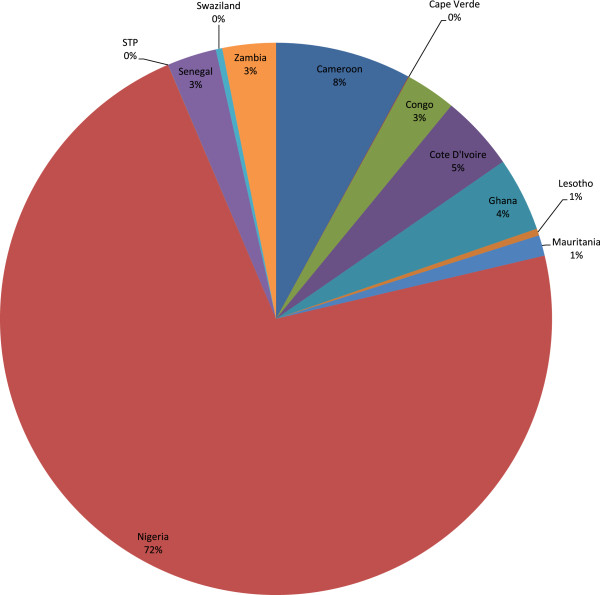
**Group 2 - Non-health GDP loss due to maternal deaths in lower middle income countries in 2010.**

The 56,482 maternal deaths in Group 2 accounted for a total non-health GDP loss of Int$ 2,001,723,471 in 2010, which was 0.34% of the total GDP for the group. The range was from Int$ 128,911 in Sao Tome and Principe to Int$ 1.4 billion in Nigeria. Figure 2 shows the distribution of the total non-health GDP loss (Int$ or PPP) across the 12 lower-middle income countries. Approximately 72.3% of the group’s loss was borne by Nigeria.

### Non-health GDP loss for Group 3 countries

**Figure 3:**
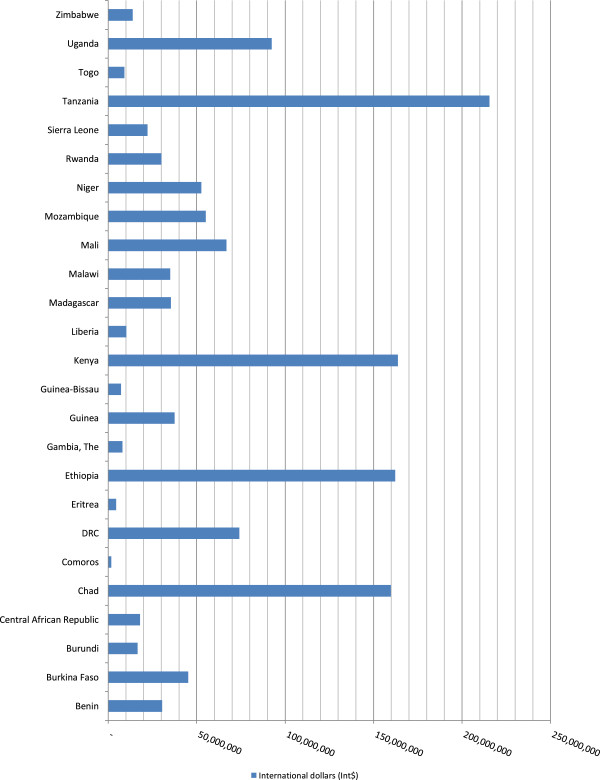
**Group 3 - Non-health GDP loss due to maternal deaths in low income countries.**

The 83,409 maternal deaths in Group 3 resulted in a total non-health GDP loss of Int$ 1,367,677,434 in 2010. This was 0.29% of the group’s total GDP. The total cost varied from Int$ 1.8 million in Comoros to Int$ 215.5 million in Tanzania. Figure 3 portrays the distribution of the total non-health GDP loss (Int$ or PPP) across the 25 low income countries in Group 3. Chad, Ethiopia, Kenya and Tanzania combined accounted for almost 51.3% of the loss.

### Average non-health GDP losses

Table 5 shows the averages for non-health GDP loss per maternal death and for GDP loss per person in the population. The average non-health GDP loss per maternal death was obtained by dividing a group’s total productivity loss by its total maternal deaths. The average GDP loss per person in the population was calculated by dividing a group’s total non-health GDP loss by its total population (see Table 4)*.*

The average non-health GDP lost per maternal death ranged between Int$ 16,397 for Group 3 and Int$ 139,219 for Group 1. The average non-health GDP lost per person in the population varied from Int$ 2.9 in Group 3 to Int$ 9.6 in Group 1. The average non-health GDP lost per maternal death in Group 1 was almost fourfold that of Group 2 and almost ninefold that of Group 3.

One of the main drivers of productivity loss is the size of per capita GDP. For example, even though middle income countries like Botswana, Gabon, Mauritius and Namibia had relatively low maternal death levels of 75, 94, 10 and 120, respectively, their indirect costs per maternal death were very high, at Int$ 292,394, Int$ 27,7221, Int$ 314,000 and Int$ 134,393 respectively. On the other hand, low income countries such as Burundi, Democratic Republic of Congo, Liberia and Zimbabwe with relatively high maternal deaths of 2200, 15,000, 1200 and 2200 had comparatively low productivity losses per maternal death of Int$ 7504, Int$ 4940, Int$ 8542 and Int$ 6260, respectively.

### Sensitivity analysis

A discount rate of 3% was used in our study, since that is what is commonly used in WHO’s global burden of disease estimates and health systems’ performance assessment work. It is also the rate used in economic evaluations of developing countries. Table 5 presents the results from one-way sensitivity analysis that was conducted at 5% and 10% discount rates to ascertain their effect on non-health GDP loss estimates.

Using the discount rate of 5% reduced the grand total non-health GDP loss by Int$ 851,548,458 (19.1%) and the average cost per maternal death by Int$ 5,764. Application of the 10% discount rate decreased the grand total non-health GDP loss by Int$ 2,127,747,182 (47.7%) and the average cost per maternal death by Int$ 14,402. This shows that the magnitude of the total economic loss is also dependent on the discount rate used.

### Limitations of the study

This study has a number of limitations. First, cost-of-illness studies such as the one reported in this paper are not a basis for setting public health priorities but rather a way of highlighting the economic losses resulting from maternal deaths to divert from the traditional epidemiological assessment of morbidity and mortality [24–27]. Therefore, the purpose of our study was not to guide priority setting but rather to raise the awareness of the public and policy-makers in the health and finance sectors of the losses in non-health GDP that maternal deaths could generate in the Region.

Second, our study is a partial cost-of-illness analysis, since it did not include (a) morbidity costs due to absenteeism from work and cost of time lost by family members accompanying pregnant women for delivery; and (b) direct health care costs that might be incurred addressing maternal complications before death occurs [28]. In addition, the cost of pain and suffering sustained before death occurs and the grief among family members for losing a loved daughter, mother, spouse, sister, sister-in-law etc. were omitted. Unfortunately, the implicit assumption is that the economic value of intangible losses is zero, which many people, including, the authors would contest. We did not include the data on this variable, which can be obtained only through willingness-to-pay surveys [29–32].

Third, the cost-of-illness approach is a static model analysis that does not consider the effects of maternal deaths on capital accumulation, investment in human capital, demographic changes and diminished economic growth [11, 14].

Fourth, contributions of housewives are not captured in GDP calculations. A sizeable proportion of women in the Region are housewives, whose contributions are not included in the national income accounts, including GDP calculations. It might be this kind of omission that erroneously led to limited attention to maternal health services and inadequate allocation of requisite resources for provision of services that would have saved the lives of many women. In other words, women continue to pay the price for weaknesses in methodologies for national income accounting, including GDP calculations. It is was for that reason that we consciously chose to apply GDP per capita for all maternal death cases irrespective of whether their contributions to human development were marketed or not. The fact that no investment has been made to quantify the monetary value of housewives’ contribution to human development or even to GDP in a narrow sense is no reason to neglect or ignore them. As UNDP states, all too often, women and girls are discriminated against in health, education and the labour market – with negative repercussions for their freedoms [33].

Fifth, we did not adjust our estimates for labour participation and employment rates. Critics may argue that not every woman who died from a pregnancy or childbirth related cause or within 42 days after delivery would have worked or been productive if she had not died. Given that women contribute in multiple ways to both marketed and non-marketed human development, we refrained from the temptation to adjust our estimates for labour participation and employment rates [28].

Sixth, the use of average per capita GDP masks the income and human development inequalities that exist among various countries in the Region [33].

Lastly, it is common knowledge that vital registration systems for births and deaths in many countries in the African Region are weak. That is why even the maternal mortality estimates contained in the WHO, UNDP, UNICEF and World Bank reports are projections based on a battery of second-best methods, such as sisterhood methods. Weak routine information systems, inadequate registration processes and reliance on periodic household surveys as the main source of population-based data are all familiar obstacles to improving public health in poor countries [34]. Other reasons that make it difficult to accurately measure maternal mortality are that a woman’s pregnancy status may not be known at death and that in country settings where medical certification of cause of death does not exist, accurate attribution of female deaths as maternal death is difficult [35].

## Conclusions

Despite the limitations, the estimates reported in this paper demonstrate that maternal mortality imposes a sizeable economic burden on African Region countries. Majority of the maternal deaths and the associated economic losses could have been averted if the following cost-effective mix of interventions for maternal and neonatal health were readily accessible to those who need them [36–42]:

-  Community-based newborn care package, including support for breastfeeding mothers and for babies with low birth weight and community-based management of neonatal pneumonia
-  Antenatal care, including tetanus toxoid shots
-  Screening for and treatment of asymptomatic bacteriuria and syphilis
-  Skilled maternal care and immediate care of newborn
-  Treatment of severe pre-eclampsia
-  Management of obstructed labour, breech presentation and fatal distress
-  Steroids for preterm births
-  Management of maternal sepsis
-  Antibiotics for preterm rapture of membranes
-  Referral for postpartum haemorrhage

What is needed to save lives is quality care before, during and after childbirth; essential medicines such as antibiotics and oxytocin; safe blood supplies; contraception and safe abortion services; and surveillance to ensure that every death is counted and its cause recorded [43–47].

The heavy economic burden and human rights considerations associated with maternal mortality underscore the urgent need for scaling up women’s health investments to fully implement the resolutions on women’s health from the African Union [48, 49], the WHO Regional Committee for Africa [10, 50], the World Health Assembly [51–54] and the United Nations [55–62], and specifically resolutions on prevention of maternal morbidity and mortality.

## Authors’ information

JMK holds a PhD in economics from the University of York, UK. He is the Programme Manager for Research, Publications and Library Services at the World Health Organization’s Regional Office for Africa, Brazzaville, Congo. Between 2005 and 2010 he was Programme Manager for Health Financing and Social Protection with that office. Prior to joining WHO in 1999, he was a senior lecturer and coordinator of health economics master’s degree programme at the University of Cape Town, South Africa. He has edited two health economics books, *Economic evaluation of public health problems in sub-Saharan Africa* and *Efficiency of health system units in Africa: a data envelopment analysis*.

GMM is Professor of Economics at the School of Economics, University of Nairobi, Kenya. He holds a PhD in economics from Boston University, USA. He was Chairman of the Economics Department and Dean of Commerce at Kenyatta University 1989–1993 and later Chairman of the Department of Economics at the University of Nairobi 2003–2005. He has edited six books: *Reproductive health, economic growth and poverty in Africa*, *Poverty in Africa: analytical and policy perspectives*, *Decentralization and devolution in Kenya*, *Malaria and poverty in Africa*, *Improving health policy in Africa* and *Social provision in low-income countries: new patterns and emerging trends*.

JNO has MD and MSc degrees in health economics from the University of York, UK. She is currently the Regional Advisor on health systems partnerships, monitoring and evaluation at the WHO Regional Office for Africa. Prior to this, she worked with WHO for over 10 years at the country level in Uganda and supporting other countries in the Region on different aspects of health systems strengthening. She also undertook research on different aspects of health economics and health financing. Over the years, she has published on health systems performance assessment, health financing, health and poverty, economic evaluation and knowledge translation.

RDKM is currently pursuing her degree in psychology at the United States International University, Nairobi, Kenya. She has worked as a research assistant to JMK in a number of projects.

## Electronic supplementary material

Additional file 1: **Data inputs.** (PDF 249 KB)
