# Supplementary material for: Indirect cost of maternal deaths in the WHO African Region in 2010
Source: BMC Pregnancy Childbirth. 2014 Aug 31;14:299. doi: 10.1186/1471-2393-14-299 (PMC4164751; doi:10.1186/1471-2393-14-299)
Supplement: Supplementary file 1 — Additional file 1: Data inputs. (PDF 249 KB) [file 12884_2014_1181_MOESM1_ESM.pdf]

ADDITIONAL FILE 1: DATA INPUTS.

The file contains Table 1: Female Life Expectancy At Birth In Countries Of The Who African Region In 2011; TABLE 2: Per capita GDP, Per capita total expenditure on health, and Non-health GDP per capita, PPP (current international $) in 2010; and Table 3: Total Maternal Mortality In 2010

**TABLE 1: FEMALE LIFE EXPECTANCY AT BIRTH IN COUNTRIES OF THE WHO AFRICAN REGION IN 2010**	
**Country**	**Life expectancy**
Algeria	74
Angola	53
Benin	59
Botswana	67
Burkina Faso	57
Burundi	54
Cameroon	54
Cape Verde	76
Central African Republic	50
Chad	53
Comoros	63
Congo	59
Côte d'Ivoire	58
Dem. Rep. of the Congo	51
Equatorial Guinea	55
Eritrea	64
Ethiopia	62
Gabon	64
Gambia	60
Ghana	65
Guinea	56
Guinea-Bissau	52
Kenya	61
Lesotho	50
Liberia	60
Madagascar	68
Malawi	58
Mali	53
Mauritania	60
Mauritius	78
Mozambique	53
Namibia	66
Niger	57

Nigeria	54
Rwanda	61
Sao Tome and Principe	65
Senegal	62
Seychelles	78
Sierra Leone	47
South Africa	60
Swaziland	51
Togo	58
Uganda	57
United Republic of Tanzania	61
Zambia	56
Zimbabwe	55

Source: WHO [22]

TABLE 2: Per capita GDP, Per capita total expenditure on health, and Non-health GDP per capita, PPP (current international $) in 2010

**Country**	(A) Per capita GDP (Int$)*	(B) Per capita total expenditure on health (PPP int. $) in 2010**	(C=A-B) Non-Health PCPGDP (Int$)
Algeria	7,975.80	364.28	7,611.52
Angola	5,554.07	194.05	5,360.02
Benin	1,453.70	69.82	1,383.88
Botswana	14,290.18	711.50	13,578.68
Burkina Faso	1,325.23	91.99	1,233.24
Burundi	523.03	54.26	468.77
Cameroon	2,153.39	122.16	2,031.23
Cape Verde	4,421.81	167.37	4,254.44
Central African Republic	958.79	29.88	928.91
Chad	2,005.57	59.78	1,945.79
Comoros	1,163.72	57.92	1,105.80

Congo, Democratic Republic of	369.32	26.26	343.06
Congo, Republic of	4,123.90	96.91	4,026.99
Côte d'Ivoire	1,937.90	114.67	1,823.23
Equatorial Guinea	28,186.96	1,395.46	26,791.50
Eritrea	493.01	17.14	475.87
Ethiopia	967.15	50.04	917.11
Gabon	14,094.77	531.80	13,562.97
Gambia, The	1,940.56	89.89	1,850.67
Ghana	1,635.28	85.25	1,550.03
Guinea	987.94	67.32	920.62
Guinea-Bissau	1,138.68	81.86	1,056.82
Kenya	1,618.53	72.10	1,546.43
Lesotho	1,762.61	183.78	1,578.83
Liberia	541.93	88.04	453.89
Madagascar	934.22	34.55	899.67
Malawi	723.65	74.10	649.55
Mali	1,204.88	69.11	1,135.77
Mauritania	2,321.69	137.93	2,183.76
Mauritius	13,509.28	835.46	12,673.82
Mozambique	885.74	57.30	828.44
Namibia	6,702.61	360.00	6,342.61
Niger	705.85	36.00	669.85
Nigeria	2,387.56	128.00	2,259.56
Rwanda		120.49	1,041.20

	1,161.69		
São Tomé and Príncipe	1,698.38	150.64	1,547.74
Senegal	1,845.76	111.15	1,734.61
Seychelles	22,708.71	805.63	21,903.08
Sierra Leone	1,096.43	171.30	925.13
South Africa	10,470.26	914.52	9,555.74
Swaziland	5,195.10	410.69	4,784.41
Tanzania	1,417.65	100.19	1,317.46
Togo	948.44	74.00	874.44
Uganda	1,243.03	117.02	1,126.01
Zambia	1,507.83	91.67	1,416.16
Zimbabwe	435.57	56.00	379.57

Sources: *The World Bank [21]; **World Health Organization [4]

TABLE 3: TOTAL MATERNAL MORTALITY IN 2010	
Country	Number of maternal deaths
Algeria	690
Angola	3,600
Benin	1,200
Botswana	75
Burkina Faso	2,100
Burundi	2,200
Cameroon	4,900
Cape Verde Is	8
Central African Rep	1,400
Chad	5,300
Comoros	79
Congo	800
Cote d'Ivoire	2,700
DRC	15,000
Equatorial Guinea	61

Eritrea	460
Ethiopia	9,000
Gabon	94
Gambia The	230
Ghana	2,700
Guinea	2,400
Guinea Bissau	460
Kenya	5,500
Lesotho	370
Liberia	1,200
Madagascar	1,800
Malawi	3,000
Mali	3,800
Mauritania	590
Mauritius	10
Mozambique	4,300
Namibia	120
Niger	4,500
Nigeria	40,000
Reunion	
Rwanda	1,500
Sao Tome et Principe	4
Senegal	1,700
Seychelles	
Sierra Leone	2,000
South Africa	3,200
Swaziland	110
Tanzania Uni Rep	8,500
Togo	580
Uganda	4,700
Zambia	2,600
Zimbabwe	2,200

Source: WHO, UNICEF, UNFPA and The World Bank [2]
